# Compositions and Microstructures of Carbonated Geopolymers with Different Precursors

**DOI:** 10.3390/ma17071491

**Published:** 2024-03-25

**Authors:** Zhuguo Li, Ko Ikeda

**Affiliations:** Graduate School of Science and Technology for Innovation, Yamaguchi University, 2-16-1 Tokiwadai, Ube 755-8611, Japan; k-ikeda@yamaguchi-u.ac.jp

**Keywords:** geopolymer, carbonation, compositional change, microstructure, fly ash, slag, ternary diagram analysis

## Abstract

It is thought that geopolymers are easy to carbonate, especially when they are cured in ambient temperatures. Matrix gel’s composition and microstructure, and new products of geopolymers (GPs) after carbonation were investigated in this study on the basis of XRD and SEM-EDS measurements and ternary diagram analysis, which were prepared from low-lime fly ash (FA) and ground granulated blast-furnace slag (GGBS) alone or a blend, as a precursor. The specimens were hardened in a 20 °C environment with alkali activator solution (S/N = 1.1 in mole), followed by storage under sealing or accelerated carbonation. XRD patterns show that carbonation products were nahcolite for the sole FA-based GP and calcite for the GPs using GGBS alone or as a blend. The SEM images of carbonated samples show that there were cube-shaped calcite and small calcite particles in the GGBS-based GP, but hail-like particles in the FA/GGBS blend-based GP. The hail-like particles were complexes of calcite and C-A-S-H gels determined by ternary diagram analysis, and were found to plug the top of the pores of the spongy C-A-S-H gels. We also confirmed that combined ternary diagram analysis of S-(C + M + N)-A and A-(C + M)-N are very effective in determining the gel type of a geopolymer, as well as the products and compositional changes after carbonation, in which oxide components of gels are determined by SEM-EDS. In the former diagram, C-A-S-H gels were plotted linearly along the (C + M + N)-albite (Ab) join, while N-A-S-H gels showed a scattered distribution. In the latter diagram, the plots for N-A-S-H and C-A-S-H gels are distributed in different zones. N = Na_2_O, C = CaO, M = MgO, A = Al_2_O_3_, S = SiO_2_, H = H_2_O.

## 1. Introduction

Global warming has been an urgent issue for mankind since the end of the last century. One of the causes of global warming is considered to arise from carbon dioxide, known as a “carbon footprint”. The cement industry, which relies on limestone, is no exception in the effort to reduce carbon emission. As one of many countermeasures, the direct capture of CO_2_ from the exhaust is attempted, followed by recycling CO_2_, e.g., to produce methane together with hydrogen, but currently the development of this technology remains a challenge. Under this circumstances, geopolymers (GPs) may have the potential to be a solution for the cement industry. GPs are characterized by their slight superiority to Portland cement (PC) currently used. Due to non-clinkering processes and the absence of raw limestone, GP has a lower carbon footprint. CO_2_ emissions could be reduced by 80% for metakaolin-based GP, compared to PC [1]. On the other hand, the CO_2_ emissions of FA (70%)/GGBS(30%) blend-based GP concrete are 40–60% of PC concretes under the conditions that the two kinds of concrete have almost the same slump (about 15 cm) and compressive strength (about 30 MPa) [2]. In addition, GP has some other advantages such as early strength development, high alkali-silica reaction resistance (ASR-resistivity), high acid resistance, high fire resistance, massive recycling of wastes, and immobilization of toxic chemical species, etc.

The solidification of geopolymer is attributed to the polycondensation of the [Si(OH)_4_]-monomers that are derived from precursors and alkali solution such as NaOH aqueous solution or/and silicate solution. These solutions, called “activators” play a role in the quick dissolution of aluminosilicate precursors called “active fillers”. The precursors supply foreign ions such as Al^3+^ and Si^4+^ into activator solutions. Then, polycondensation of activators is triggered by these foreign ions to solidify themselves into insoluble monoliths as a result of gel formation.

A typical precursor is metakaolin (MK) obtained from kaolin through calcination. MK-based geopolymers, using potassium-based activators, can be regarded as the first generation geopolymer that is characterized as calcium-free. Na-based analogues were also intensively studied, for instance [3,4]. However, kaolin resources are unevenly distributed throughout the world. Fly ash (FA), which is a kind of waste discharged from coal power plants, has attracted attention as an alternative to metakaolin. Simultaneously, sodium silicate and/or caustic soda solutions are generally used as activators. Although sole FA-based geopolymers with high strengths were reported [5,6,7], these types of GPs exhibits generally low strength when cured at room temperature [8]. To address this issue, ground granulated blast-furnace slag (GGBS) is conveniently blended to achieve a high strength even at room temperature. According to the literature, the optimal blending ratio (%) of FA/GGBS is 70/30 by mass [9]. It should be noted that these types of GPs are now at the practical application stage [10,11]. It is generally considered that the binders generated in MK or FA-based GP are N-A-S-H gels where aluminums replace tetrahedral silicones within the 3D tectosilicate structure, i.e., Si^4+^→Al^3+^ + M^+^, where M^+^ is alkali ion such as Na^+^ and K^+^. However, for the C-A-S-H gels derived from GGBS, aluminums replace silicones to link the tetrahedra with the drierketten 1D inosilicate structure [1]. Counterpart alkalis enter into spacious interlayers in the structure.

Rayment, in 1982, studied aluminum incorporation into C-S-H gel for the first time, initially written as C-(A)-S-H where the brackets represent small amounts [12,13]. Since then, the compositions and structure of this new type of gel have gradually been clarified with the advent of solid state nuclear magnetic resonance spectroscopy (solid state-NMR), primarily the magic angle spinning type (MAS-NMR). Currently, two models are proposed for the structure of C-A-S-H gel. One is the “single-chain model” or the “omission drierketten model” [13,14,15,16,17,18,19,20,21] which is a traditional model. The other is the “double-chain model” or the “cross-linked model [22,23,24]. The bridging tetrahedra enables coupling across the so-called interlayer space, where -Al-O-Al- coupling is prohibited [25,26]. Other plausible models are represented in the literature [27,28], in which extra chemical species are accommodated in an interlayer space such as Ca^2+^, Na^+^, H^+^, and H_2_O. These species play a role of maintaining charge neutrality. Thus, the classic concept of replacement, e.g., Si^4+^→Al^3+^ + M^+^ (alkalis) is diversified at the moment. It is reported that GGBS-based GP, otherwise known as alkali-activated material (AAM) activated by NaOH solution, gives rise to a single-chain model, while the activation of a sodium-silicate solution yields the hardening of GGBS in the double-chain model [28,29].

Understanding how to determine the compositions and type of GP gels is not easy, since the gels are almost amorphous. Therefore, X-ray diffractometry (XRD) is ineffective. Fourier transform infrared spectroscopy (FTIR), solid-NMR, X-ray photoelectron spectroscopy (XPS), and scanning electron microscopy equipped with energy dispersive X-ray spectrometer (SEM-EDS), etc., are most conveniently used at present.

The results of SEM-EDS point analysis about GPs are often used to plot the ternary diagram of SiO_2_-CaO-Al_2_O_3_ [30,31,32,33], but for determining Na_2_O behavior, this kind of ternary diagram is insufficient, thus a more complicated quaternary diagram was introduced to add the Na_2_O component [32]. If the gels are Na-bearing C-A-S-H, it is expressed as C-(N)-A-S-H [24]. Otherwise, if the gels are Ca-bearing N-A-S-H, it is expressed as (N,C)-A-S-H [32] or N-(C)-A-S-H [34]. The N-(C)-A-S-H and C-(N)-A-S-H gels, generally abbreviated to N-A-S-H and C-A-S-H, exhibit plane-like and sponge-like textures, respectively [35]. Therefore, distinguishing between the two gels is possible by SEM observations. In addition, the entry of calcium to pure N-A-S-H has already been confirmed by MAS-NMR and FTIR techniques [36], resulting a tectosilicate structure for the Ca-bearing N-A-S-H gel.

Geopolymers are reported to be easily carbonated, compared to PC [37]. The carbonation of pore solutions and gels results in different products for different types of gel and different residual alkali-activator in pores, and also may change the microstructures of the gels. However, the compositional and microstructural changes in carbonated GP are not fully understood at present.

In this paper, we investigate the compositions, microstructures, gel type, and carbonation products of geopolymers prepared from different blends of FA and GGBS before and after accelerated carbonation, based on the results of XRD and SEM-EDS analyses. The main objectives are as follows: First, the identification of carbonation products and the differences between GPs with different GGBS blending ratios by XRD analysis. Second, the clarification of gel morphologies and compositions, as well as changes after carbonation for different GPs using SEM-EDS analysis. Third, the interpretation of gel’s compositions and changes due to carbonation on the basis of SEM-EDS results by the two ternary diagrams, SiO_2_-(CaO + MgO + Na_2_O)-A1_2_O_3_ and Al_2_O_3_-(CaO + MgO)-Na_2_O, of which the uses in pair have already been confirmed to be an effective method for judging the compositional feature of GP gels in previous studies [35].

## 2. Materials and Methods

### 2.1. Sample Preparation and Carbonation

Low-lime coal fly ash (FA) and ground granulate blast-furnace slag (GGBS) were used as precursors of geopolymers in this study, of which chemical compositions are represented in Table 1 together with densities and Blaine specific surface areas. The FA and GGBS meet the JIS class II (>250 m^2^/kg, Blaine) and the JIS grade 4000, respectively. The chemical compositions were determined by X-ray fluorescence spectroscopy (XRF) analysis employing ZSX101e that was manufactured by Rigaku Corporation in Tokyo, Japan.

In this study, stock solutions of sodium disilicate and caustic soda were firstly prepared from commercially available sodium disilicate and caustic soda solutions by diluting with deionized water, of which the specifications are as follows: S/N 2.1 in molar, and density 1.27 kg/dm^3^ for the former, and 10 mole/L, and 1.33 kg/dm^3^ for the latter, respectively. Then, an alkali-activator solution, hereafter called GP-liquor, was prepared by mixing both solutions in a 3:1 by volume ratio, the characteristics of which are as follows: density 1.31 kg/dm^3^ and concentration 26.3% by mass with S/N 1.1 in molar. Mix proportions of GP pastes are shown in Table 2 together with the characters of the GP-liquor used.

A Hobart planetary mixer was used to mix the three GP pastes. The premix procedure was skipped for a neat mixture using sole FA or sole GGBF as a precursor. The fresh pastes were filled into cylindrical molds of 50 mm in diameter and 100 mm in height, and then compacted with a table vibrator. Two specimens were prepared for each mixture. All the paste specimens were sealed with a plastic film for preventing water loss to reduce shrinkage and to avoid natural carbonation in the air, then cured in a room regulated at 20 °C and 60% R.H. for 28 days. Demolding and then sealing with the film was conducted at 3 days. After 28 days, half of them were unsealed and moved into an accelerated carbonation apparatus, ACT-250 made by Asahikagaku Ltd. in Tokyo, Japan, regulated at 20 °C, 60% R.H., and 10% of CO_2_ concentration for 35 days. Other specimens continued to be stored in the curing room for 35 days, kept with wrap sealing. Finally, after 35 days accelerated carbonation or continuous curing was reached, the alkalinity of each specimen was checked by spraying a phenolphthalein solution indicator. Then, samples were collected from the specimen’s surface layer to detect carbonation products. It was thought that the specimens, cured for 28 days and then stored for 35 days in the sealed state, were not carbonated. For more details, refer to the previous study [38].

### 2.2. XRD Analysis

**Figure 1:**
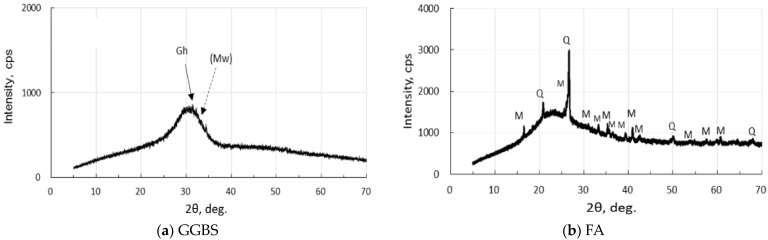
XRD diagrams of precursors: (**a**) GGBF, and (**b**) FA. [Notes] Gh: gehlenite, (Mw): merwinite (very few), Q: quartz, M: mullite.

XRD analysis was carried out, employing MiniFlex 300/600 made by Rigaku Corporation in Tokyo, Japan. The operating conditions are as follows: 40 kV-15 mA X-ray tube power, doubly Ni-filtered CuKα radiation, 4°/min-0.01° step scan, 1.25°-10 mm-13 mm-13 mm slit system, and 5–70°, 2θ range. XRD diagrams of the active fillers are represented in Figure 1. GGBS has few crystals, and FA contains quartz and mullite in addition to amorphous phases. There were few iron-bearing minerals in two fillers.

### 2.3. SEM-EDS Analysis

SEM-EDS analysis was carried out by employing JSM-7600F equipped with a EDS spectrometer made by Jeol Ltd. in Tokyo, Japan. All the images were taken under 15 kV accelerating voltage. Outputs of point analytical data were automatically corrected by ZAF. Sample pieces just after 28 days of curing were polished with silicon carbide sand papers, #400, #800, and #1500, then finished with corundum sand paper, #3000. Water was used as a polishing media throughout. Finally, so-called “shallow polished samples” were prepared by finishing with ultrasonic cleaning. After air-drying, the samples were coated with platinum/gold. By regulating the polishing duration, so-called “deep polished samples” can also be prepared, which corresponds to mirror surface finishing. However, this is out of the scope of this study. In order to observe micro-textures, “shallow polished samples” are more suitable, since focusing every inch of the image becomes easy without destroying the original 3D-texture of the surface, particularly with C-A-S-H gels possessing sponge-like micro-surface textures. The SEM-EDS point analysis data in atomic percent (Ca, Mg, Si, Al, Na, etc.) were recalculated into oxide components (CaO, MgO, SiO_2_, Al_2_O_3_, Na_2_O, etc.) in molar.

## 3. Results and Discussion

### 3.1. Carbonation

#### 3.1.1. Method of XRD Identification

Prior to identifying the XRD peaks of GPs, XRD patterns of C-S-H should be mentioned. It is well-known that tobermorite-like peaks appear for synthetic semi-crystalline C-S-H(I) possessing a wide compositional range, experimentally 0.5–1.5 and theoretically 0.66–1.5 C/S in molar [39,40,41,42]. Only (001), (220), (400), and (040) reflections of orthorhombic setting are the focus of this discussion. According to our understanding of hydrated PC paste, however, (220, d = 0.307 nm), (400, d = 0.280 nm), and (040, d = 0.183 nm) peaks are generally recognized as broad peaks, and basal reflections of (001, d = 1.25 nm) is generally absent. Reflections of (220) and (400) are apt in bundle due to broadness. This product is originally called “tobermorite gel” [43,44], but nowadays is often called “C-S-H”. C-S-H is not crystalline but semi-crystalline, only showing broadened peaks. 

It should be further noted that the strongest (220) reflection of C-S-H overlaps with the strongest (10·4) reflection of calcite of hexagonal setting, but discriminating between them is not difficult since calcite exhibits a sharp peak. Moreover, 2θ-positioning of (040) of C-S-H is different from (10·8) and (11·6) of calcite. Therefore, we can detect calcite formation for the carbonated GP samples by only checking whether they have (10·8) and (11·6) reflections in the XRD patterns. In addition, it should be noted that the third strongest reflection of (11·2) of quartz is overlapped with (040) reflection of C-S-H [45]. According to Refs. [20,46,47], what is stated just above is applicable to Al-bearing C-S-H, i.e., C-A-S-H with a single-chain structure. Furthermore, it was reported that NMR spectra of Q^3^ (1Al) peculiar to the gels with double-chain structure are not observed in the carbonated FA/GGBS blend-based GP [48]. Thus, the discussion of the XRD patterns of GPs was performed on the basis of the single-chain structure model in this study.

#### 3.1.2. Results of XRD Identification

As seen in Figure 1, the XRD pattern of the raw GGBS sample showed a hump, a low gehlenite peak, and other unknown low peaks. However, the first strongest merwinite peak was very low and unclear, which was clearly depicted in a previous study [49]. The raw FA had quartz, mullite, and a hump due to glassy phases. 

**Figure 2:**
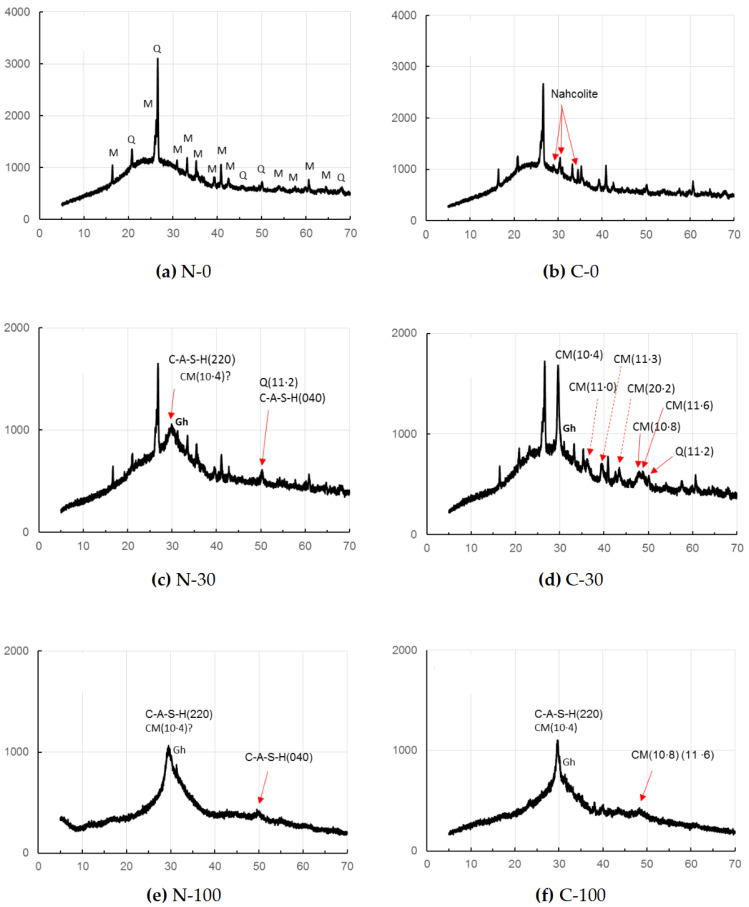
XRD patterns of GP pastes without carbonation (series N) and with the accelerated carbonation (series C). [Notes] CM: Mg-calcite, Gh: gehlenite, M: mullite, Q: quartz; ?: unclear. Solid and dashed arrows show important peaks for discussion. Axial titles omitted are the same as to Figure 1.

As seen in Figure 2, sole FA-based GP paste C-0 showed only one carbonation product after the accelerated carbonation, while no carbonate was found in the non-carbonated sample, N-0. The carbonate was identified to be nahcolite that is bicarbonate (NaHCO_3_). However, according to Ref. [48], sodium carbonate, Na_2_CO_3_∙7H_2_O, was formed rather than nahcolite in sole FA-based GP in early stage of curing, due to a high pH and wet circumstance in early age that is only 1 day. Continued polymerization and carbonation reactions over a long period of curing, leading to a decrease in the pH of C-0 may be responsible for the detection of nahcolite in C-0, since bicarbonate soda formation needs a lower pH condition, compared to carbonate soda. The hump encompassing nearly15–402θ may be due to the N-A-S-H gels duplicated with a residual glassy phase of FA.

On the other hand, for the accelerated carbonation sample, C-30, calcite formation was clearly identified as a carbonate product, while calcite was not clearly observed for the non-carbonated sample, N-30. The (10·8) and (11·6) peaks of calcite were recognized clearly for the specimen, C-30, together with strongest reflection of (10·4) of calcite. Contrary, these peaks of calcite were unclear for the non-carbonated samples, N-30, but a little calcite formation is suspected from the slightly sharp peak of (10·4).

In addition, judging from the sharpness of (11·2) reflection of quartz, the presence of (040) reflection of C-A-S-H is uncertain. The hump, encompassing nearly 15–40⁰ of 2θ, may be originated from the residual glassy phases of active fillers as well as matrix gels which will be described in detail in Section 3.2.

In the samples N-100 and C-100, using GGBS alone as precursor, (11·2) reflection of quartz was not observed due to the absence of FA. In the accelerated carbonation sample C-100, (10·8) and (11·6) of calcite peaks appeared in a bundle probably due to less calcite formation than in C-30, while these peaks disappeared in the non-carbonated sample, N-100. However, judging from the sharp peak of (10·4), calcite might form in the sample N-100. The hump observed approximately in a range of 20–40° of 2θ may be due to residual GGBS as well as C-A-S-H for both N-100 and C-100. 

No evidence of vaterite was found in this study, the presence of which was often reported so far associated with calcite [46,50], particularly with the specimens with short age, since vaterite is considered to be a metastable phase. A low gehlenite peak located at around 31⁰ of 2θ was observed, remaining intact in GGBS-bearing GP pastes regardless of carbonation. Quartz and mullite remained intact in the FA-bearing GP pastes.

**Figure 3:**
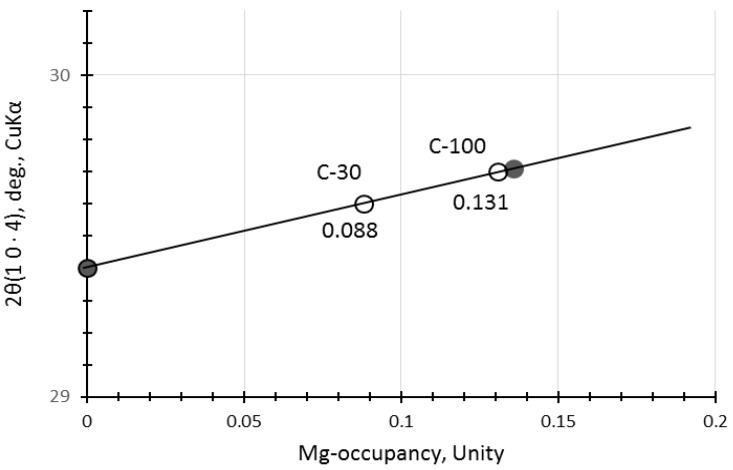
Calibration curve to determine substitution rate of Mg for Ca in Mg-calcite. [Notes] Solid circles: synthetic pure calcite and natural Mg-calcite mineral (Ca_0.861_, Mg_0.136_, Sr_0.002_)CO_3_. Open circles: the samples of this study.

The calcite may not be a pure calcite but includes some magnesium, yielding magnesian calcite (Mg-calcite) [35,51]. Hence, 2θ positions of calcite were a little bit shifted to higher angles due to contraction of different ionic radii between Ca^2+^(0.114 nm) and Mg^2+^(0.086 nm) [52,53]. The calibration curve produced from the file data (XRD cards, ICDD #01-081-2027 (synthetic calcite) and #00-043-0697 (natural Mg-calcite)) is represented in Figure 3, which shows that magnesium occupancy was reached up to 0.13 in the Mg-calcite in the accelerated carbonation samples, C-30 and C-100. Data from non-carbonated samples were omitted due to the ambiguity of calcite peak in the XRD patterns of N-30 and N-100.

### 3.2. Matrix Gels

#### 3.2.1. SEM-EDS Images and Element Maps

**Figure 4:**
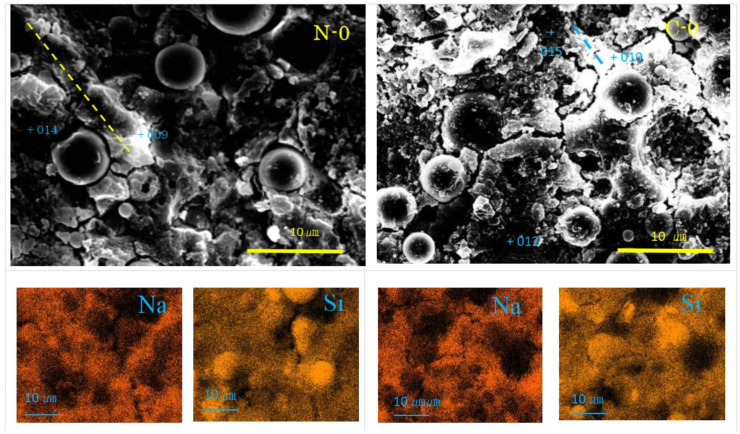
SEM-EDS images and element maps for N-0 and C-0 (Numbers represent EDS analysis spots, and typical beads texture is marked with dashed yellow or blue line).

**Figure 5:**
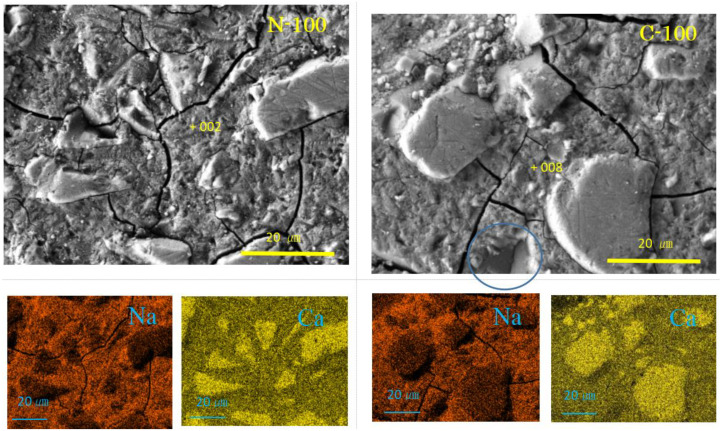
SEM-EDS images and element maps for N-100 and C-100 (Numbers represent EDS analysis spots).

**Figure 6:**
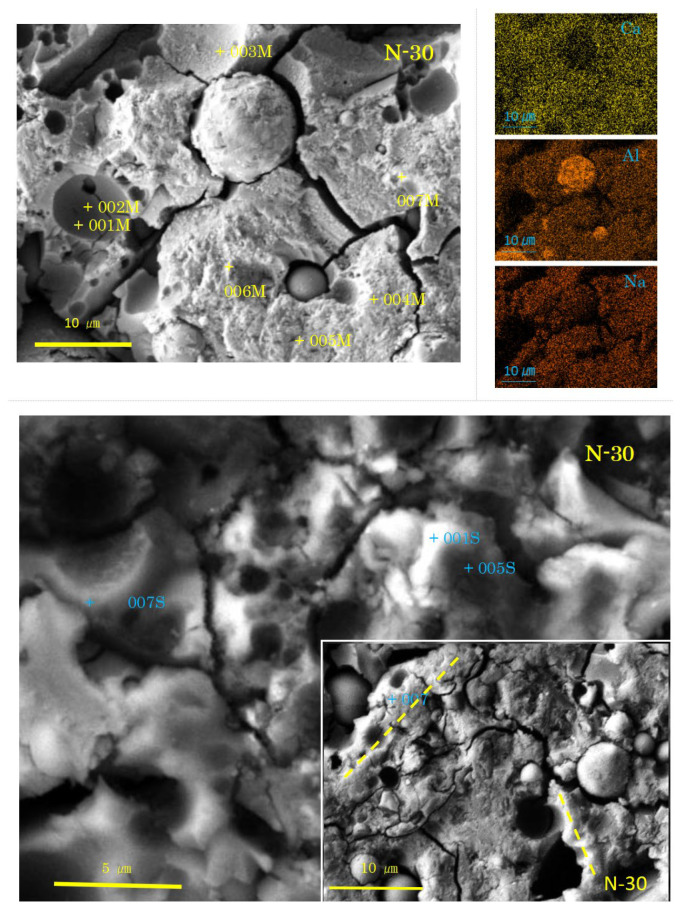
SEM-EDS images and element maps for N-30 (Numbers represent EDS analysis spots, and typical beads texture is marked with dashed yellow lines).

**Figure 7:**
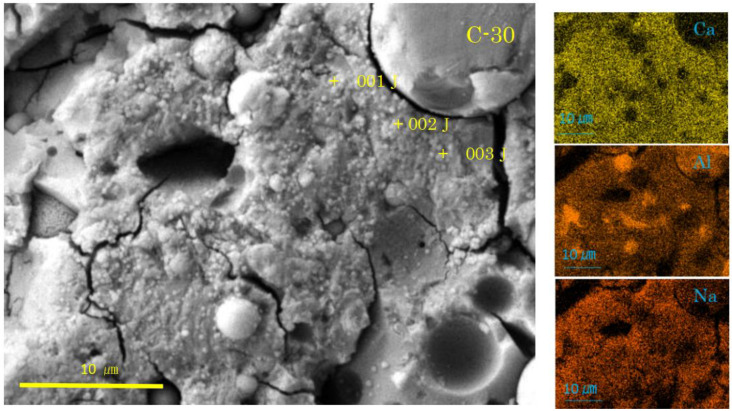
SEM-EDS images and element maps for C-30 (Numbers represent EDS analysis spots).

Selected SEM-EDS images are represented in Figure 4, Figure 5, Figure 6 and Figure 7, showing some cross sectional features. Matrix gels of N-A-S-H and C-A-S-H can be identified on the SEM images from their morphologies, flake-like gels for N-A-S-H, and sponge-like gels for C-A-S-H, regardless of carbonation. Taking account of the type of active filler, FA and/or GGBS, is helpful to identifying the gel type [35,48,54].

The cracks were formed by shocks during sample preparation. For the two extreme mixtures of sole FA and sole GGBS-based GP, only N-A-S-H or C-A-S-H was found, whereas in the intermediate mixture of FA70/GGBS30-based GP, no N-A-S-H gel was found. Three types of C-A-S-H gel were recognized in the intermediate mixture samples according to their morphologies, regardless of carbonation. The first type is here called “normal C-A-S-H” with the sponge-like texture, as observed in the sole GGBS-based GP samples. The second and the third types are here called “abnormal C-A-S-H”, which are dense gel forming pit wall, and fluffy cotton-like gel that covered the pit wall, respectively. In addition, pop-out traces of FA particles were found in the GP samples using FA, especially since there were a lot in the intermediate mixture samples.

As seen in Figure 4, Na-map and Si-map show the distribution of N-A-S-H, and the dark round areas represent residual fly ash. From the SEM images, residual FA particles can be found to have dark cores surrounded by a dissolved bright ring surface layer. However, small residual FA particles show the feature of linear alignment, called “beads” here, covered with flaky gels. One of the alignments is marked by a yellow dashed line in Figure 4, of which EDS point analysis showed it had N-A-S-H according to high Na-contents as seen in Table 3. It was reported that if GP-liquor/active filler (L/F) ratio is very large, formed N-A-S-H exhibits a sea-like plain texture due to through-solution growth [35]. Thus, the present flaky feature may be caused by the low L/F ratio in addition to the effect of filler type. 

The pores can be seen in the sample C-0, which did not form in the sample N-0, though it was reported that N-A-S-H sometimes has small pores [48,54]. Other features of the SEM images and element maps of C-0 are the same as those of N-0. However, flakes were clearly encountered in pop-out pits of FA particles. Although it is unknown at the moment whether they were N-A-S-H or quartz and mullite left after corrosion, quartz and mullite are implausible judging from their morphologies, since they generally appear as round-corroded and elongated features, respectively. Nahcolite formation was clearly identified by XRD analysis, but this carbonation product was not observed in the SEM images at the moment. This is probably because soluble nahcolite was washed away during polishing process using water media. However, according to Ref. [48], nahcolite appears as dot-like or hail-like particles, which plugs mini-pores similar to the calcite aggregates as discussed later for the sample C-30. Beads texture of N-A-S-H can be also recognized in the sample C-0. 

Figure 5 shows SEM images and element maps for the samples N-100 and C-100. C-A-S-H with a spongy texture was found, indicating the topotactic growth of C-A-S-H [35], whereas no calcite was found in the N-100. In the C-100, as observed in the top left corner of the SEM image, there are some cube-like rhombohedra less than 3 µm in size together with small particles, suggesting calcite formation based on morphologies [55,56]. Beneath the C-A-S-H layer, intact GGBS fragments can be found through a gap of a “window” marked with a blue circle.

As shown in Figure 6 and Figure 7, the SEM images of the samples N-30 and C-30 (mixture No.2) had quite different features from those of the two sole filler-based GPs (No.1, No.3). Above all, it is remarkable that there were many pits, which were the traces of dropped FA particles. 

In the sample N-30, the presence of C-A-S-H with spongy texture was determined, here called “normal C-A-S-H”. However, there was no clear proof of GGBS residues. On the contrary, two types of FA residues were recognized. The first type exhibits a rough surface of dissolved FA particles. The rough surface was easily seen on relatively large FA residues. Fingerprints of quartz and mullite were left on the internal walls of the pits of dropped large FA residues, as can be seen near the spot 003M in Figure 6. Another type of FA residue exhibits a dense and smooth surface of uniformly dissolved FA particle. The dense and smooth surface was generally present in small FA particle residues, as shown near the spot 006M. The formation of rough or smooth surface of FA residue may depend on the glassy degree of FA. The smaller the FA particle in molten state, the more vitreous in cooled state. 

Few hail-like particles were found in the sample N-30. However, many large dense pit walls swallowed small pits, which had C-A-S-H rather than N-A-S-H. At present, N-A-S-H gel was not found, as shown in Table 3. An SEM image similar to Figure 6 was reported in Ref. [57], where a large pit combines with small pits, and the small pits often align in a line. These alignments may be consistent with the “beads” found in both N-0 and C-0 (see Figure 4). Similar alignments of small pits were also found in N-30, as marked with dashed yellow lines in Figure 6. 

In the high power image, cotton-like fluffy C-A-S-H as a mantle covering the dense pit walls of FA (see the spot 001S in N-30) was observed. The pit walls (005S, 007S) and the pit bottom spots (001M, 002M, 003M) also had C-A-S-H compositions (see Table 3). There is no doubt that N-A-S-H originates from FA, and C-A-S-H from GGBS. Presumably, the C-A-S-H near FA particles has N-A-S-H gel’s compositions in the initial stage of precipitation. Subsequently, mutual diffusion may occur to incorporate these two types of gel to yield Ca-rich N-C-A-S-H, simply called C-A-S-H. Eventually, both N-A-S-H and Ca-rich C-A-S-H becomes Ca-poor C-A-S-H with low Ca/Si. This result is strongly concerned with “the incorporation issue of N-A-S-H and C-A-S-H” pointed out in some of the literature [32,33,34]. For more details, refer to Section 3.2.3, where a discussion is conducted based on ternary diagram analyses.

In the carbonated sample C-30 (see Figure 7), the C-A-S-H with sponge-like texture was observed, but not so vivid as in the non-carbonated sample N-30. Instead, there were hail-like particles, frequently plugging the top of mini-pores on the surface of spongy C-A-S-H layer. The hail-like particles were presumably calcite according to the XRD results. However, they could not be judged as calcite according to the SEM/EDS results. For example, the hail-like particles at spots 001J-003J had C-A-S-H compositions (see Table 4). Accordingly, we estimate that the hail-like particles were not entirely calcite but a complex of C-A-S-H and calcite on the basis of the ternary diagram analysis mentioned in the latter. Moreover, the plugging of mini-pores by the hail-like particles would be a cause of delayed coloring of phenolphthalein indicator to check carbonation [9,58], since the plugging may disturb the reaction of the indicator with Na-rich pore solution. Although, at the moment, no point analysis for the pits in the sample C-30 was conducted, abnormal C-A-S-H formation around the pits can be determined from the attached element maps, specifically for the pits gathered in the lower right corner (see Figure 7). These dense pits had outskirts extending outward to connect each other.

Carbonation degrees of the GP pastes were measured by thermo-gravimetry (TG-DTA), which were in the order of sole FA, FA70/GGBS30 and sole GGBS: naught, 2.9% and 1.4% for the non-carbonated sample, and 1.4%, 4.6% and 2.9% for the acceleratedly carbonated sample, respectively. Although the samples of series N were sealed, they might be slightly carbonated during demolding of GP specimens and the preparation of TG-DTA sample. The FA70/GGBS30 blend-based GP samples had a relatively high carbonation degree, probably due to more pores. As the hail-like particles of insoluble calcite block the pores, the carbonation reaction between CO_2_ and alkaline solution in the pores would become slow and come to a stop gradually with the elapsed time. It is predicted that as the blockage of pores proceeds, the carbonation rate would gradually decrease with material age. Furthermore, it was already pointed out that the traditional accelerated carbonation test method is applicable to Portland cement-based materials, but inapplicable to geopolymer for practical applications [48,59].

#### 3.2.2. Matrix Gel Compositions Detected by Point Analysis of SEM-EDS

Point analysis results are summarized in Table 3 and Table 4, respectively, for the non-carbonated and carbonated GP paste samples, respectively. Fundamentally, matrix gels were composed of CaO, Na_2_O, Al_2_O_3,_ and SiO_2_ in addition to H_2_O as main components, and contaminated with other impurities, relatively high contents for MgO and SO_3_-components, followed by P_2_O_5_ and Fe_2_O_3_-components. Manganese components are omitted due to very minor amount.

As mentioned earlier, two types of GP gel were found on the basis of morphology. N-A-S-H gel exhibits a flake-like texture with pores [48,54], whereas C-A-S-H gel shows a sponge-like texture [35]. In the intermediate mixtures, FA70/GGBS30-based GP, the presence of C-A-S-H gels were determined from the sponge feature, but N-A-S-H gels could not be found. Instead, the dense matrix gels, which were covered with fluffy cotton-like gels, were observed between the residual FA particles. According to the provisional criteria, C/N = 1.0 or (C + M)/N = 1.0 in molar ratio, that is, N-A-S-H ≤ 1.0 ≤ C-A-S-H, the dense matrix gels and fluffy cotton-like gels were not N-A-S-H but special C-A-S-H, thus called abnormal C-A-S-H in this study. Furthermore, this result is completely supported by the ternary diagram analysis mentioned later. As stated above, mutual incorporation occurring between N-A-S-H and C-A-S-H may be the reason for N-A-S-H absence. The final formation of N-A-S-H gels depends on the ratio of FA to GGBS, the boundary of which was estimated to be around FA75%: GGBS25% by mass [34]. Thus, in the present intermediate mixture (No.2) with FA70%: GGBS30%, only Na-bearing C-A-S-H was generated. FA particles remained as residues due to low dissolution in addition to compositional inhomogeneity between them [60].

The provisional criteria of compositional ratio, C/N = 1.0 or (C + M)/N = 1.0 to discriminate between N-A-S-H and C-A-S-H are basically applied to the non-carbonated samples, but there were some exceptions (spots N-0-13 and N-0-15), which were detected to possess much Fe_2_O_3_-component (see Table 3). Incorporation of iron into the tetrahedral site of GP gels was already reported [61,62]. Thus, in the non-carbonated sample, N-0, there were almost N-A-S-H gels, whereas in the N-30 and N-100, C-A-S-H gels existed according to this provisional criteria.

The provisional criteria of compositional ratio C/N = 1.0 or (C + M)/N = 1.0 for judging gel type was also basically applied to the carbonated samples. After the accelerated carbonation, each of the three types of GP paste had the same gels as the non-carbonated paste, respectively. However, spot C0-15 with rich Fe_2_O_3_ component did not meet the above provisional criteria for N-A-S-H gel.

#### 3.2.3. Ternary Diagram Analyses

Ternary diagram analyses were conducted using the data listed in Table 3 and Table 4, focusing on the major components of CaO, Na_2_O, Al_2_O_3_, and SiO_2_. MgO component was also considered since its large content was confirmed in C-A-S-H gels. The raw GGBS contained 5.32% of MgO (see Table 1). Concerning the incorporation of MgO-component into C-S-H, there have been many studies [12,63,64]. Additonally, due to the incorporation of the MgO-component, the formation of hydrotalcite or AFm is possible [17,18,35,65]. Supposing C-S-H and C-A-S-H are isostructural, it is reasonable to consider MgO in the discussion of C-A-S-H gel.

##### SiO_2_-(CaO + MgO + Na_2_O)-Al_2_O_3_ Diagram

**Figure 8:**
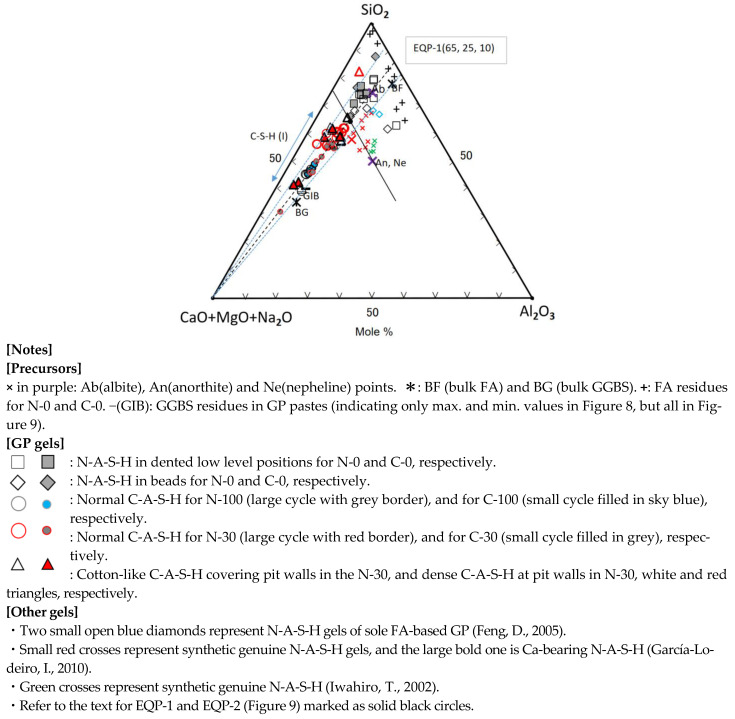
First ternary diagram based on the data in Table 3 and Table 4 [36,54,67].

**Figure 9:**
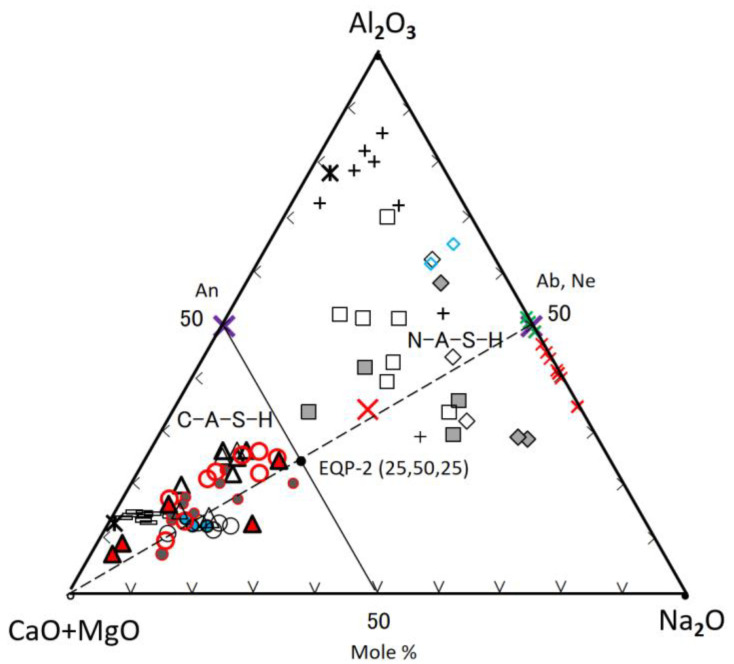
Second ternary diagram based on the data in Table 3 and Table 4, legends are the same as Figure 8.

As seen in Figure 8, most of C-A-S-H and some of N-A-S-H were plotted alongside with apical (C + M + N)-Albite (Ab) join. On the contrary, part of the N-A-S-H plots were scattered over a wide range. The reason would be inhomogeneous chemical compositions of raw FA particles, and derivative N-A-S-H gels contained the chemical compositions of raw FA particles nearby. Actually, the results of randomly performed point element analysis of individual FA residue were also scattered widely, marked with plus marks, far from the bulk composition plot (BF) of raw FA (see Figure 8). However, the plots for the GGBS residues’ compositions, marked with a black minus (GIB), were near to the plot (BG) of bulk compositions of raw GGBS particle. To avoid indistinctness caused by the overlap of many plots, only maximum and minimum composition values were plotted in Figure 8 for the GGBS residues. The omitted plots were located between maximal and minimal plots in Figure 8, but they are all represented in Figure 9 (see the minus marks in the lower left corner).

The (C + M + N)-components of C-A-S-H decreased rapidly with decreasing the blending ratio of GGBS, while the SiO_2_-component increased along the (C + M + N)-Ab join that runs close to the line connecting BF (bulk FA) and BG (bulk GGBS). In other words, it can be said that bulk compositions of FA and GGBS govern the C-A-S-H compositions in the FA/GGBS blend-based geopolymer. Silica contents in almost C-A-S-H gels are kept in the range of 40 to 66.6 mole% in terms of Si/(Ca + Si), i.e., the C-A-S-H gels still had a Ca/Si ratio = 0.5–1.5 after carbonation, which is a generally known range of Ca/Si for C-A-S-H [66]. Furthermore, distribution areas of the plots for N-A-S-H and C-A-S-H are divided by the 75S/25(C + M + N)—25(C + M + N)/75A line passing through the anorthite point (An). Almost all of the C-A-S-H plots obey this boundary line other than N-30-7. The spot N-30-7 had extraordinarily large silica content over 80% in molar (see Table 3), presumably due to the effect of residual FA particle. Nevertheless, the N-30-7 spot was plotted in the C-A-S-H area in the second ternary diagram with no doubt, of which the ternary coordinate is (25.9, 62.4, 11.7) anticlockwise from the top apex of Al_2_O_3_ (see Figure 9). In addition, there are some plots in the acceleratedly carbonated C-30 samples deviating from the C-30 group located in the central of the trend line. In these plots, specifically in C-30-1F spot, the formation of a complex of C-A-S-H and calcite is suggested.

Al_2_O_3_ incorporation to Na-bearing C-A-S-H reaches 7–11 mole%, 9 mole% in average [31]. In present study the value was nearly 5–12 mole%, 9 mole% in average too. On the other hand, Al_2_O_3_ content is 6–13 mole% for Na-free C-S-H, 9.5 mole% in average [30], being slightly larger than C-A-S-H. Therefore, Al_2_O_3_ incorporation into C-A-S-H would be limited to relatively small amounts, regardless of alkali presence and carbonation.

The compositions of normal C-A-S-H, formed in the sole GGBS-based GP pastes (N-100 and C-100) after carbonation were plotted near to the composition plot (BG) of bulk GGBS. The plots for the normal C-A-S-H in the FA/GGBS blend-based GP paste (N-30) without carbonation were shifted toward the Ab point, and located at intermediate positions of the trend line. However, some composition plots for the acceleratedly carbonated GP paste (C-30) using FA/GGBS blend returned from the intermediate positions toward to apical (C + M + N). As a result, these plots fall into the areas of C-100 and N-100 plots. From Figure 8, we found two types of C-S-A-H to have their composition return toward to apical (C + M + N). The first one was the hail-like C-A-S-H gel in the sample C-30, and the second one was the normal C-A-S-H gel on the pit walls in the sample N-30 (see Table 3 and Table 4 and Figure 6 and Figure 7). However, the latter is not a real composition return, as explained later.

Refs. [68,69] reported that synthetic C-S-H decomposes partially into Ca-carbonate, CaCO_3_ in the TG-DTA analysis, and the C-S-H gels with Ca/Si = 0.75 (42.9% Ca/57.1% Si) and 0.67 (40.1% Ca/59.9% Si) are more resistant to carbonation, i.e., the calcium-rich C-S-H gels are more easily carbonated than the calcium-poor ones. However, the type of calcium carbonate is actually unknown since CaCO_3_ formation was not determined by XRD in the two studies. On the other hand, amorphous Ca-carbonate, aragonite, and vaterite were identified by Raman spectroscopy [70,71], whereas only vaterite and calcite formations were reported [48,72]. In this study, however, XRD data clearly showed the incorporation of calcite into C-A-S-H in the C-30 sample with the accelerated carbonation, as shown in Figure 2. Accordingly, FA/GGBS blend-based GP with Ca-poor C-A-S-H gels are easier to be carbonated, which contradicts the carbonation behavior of C-S-H gels. Therefore, the general knowledge about the carbonation behavior of C-S-H in hardened PC may not be directly applicable to the C-S-H gels in geopolymers, and thus further investigation is needed on this issue. 

The most probable reason of easier carbonation of FA/GGBS blend-based GP may be due to its more porous structure, compared to the sole GGBS-based GP with rich-Ca, as pointed out by Ismail et al. [34]. This recognition was also confirmed by the SEM images of the sample C-30 in this study (see Figure 7). Obviously, the composition movement of C-A-S-H toward to apical (C + M + N) after carbonation requires extra CaO-component. Although calcite formation was not clearly observed from the SEM images at the moment, we suppose that the hail-like C-A-S-H gels are complexes of C-A-S-H and calcite crystals. The extra CaO-component may come from the GGBS residues, which dissolved continuously in the pores with remaining alkaline solution. The formation mechanism of calcite in the hail-like C-A-S-H gels may be the same as that of stalactite growth. Preexisting C-A-S-H gels are also a potential source of calcite.

The spots 001M and 002M in the pit walls of the non-carbonated sample N-30, as shown in Figure 6, were recognized to have the composition return toward to apical (C + M + N) from the intermediate positions of the trend line, as shown in Figure 8. The occurrence of composition return in the non-carbonated GP is difficult to interpreted. This would be attributed to heterogeneous compositions of raw FA particles, i.e., the difference in the silica dissolution from FA. which determines the compositions of precipitated gels, i.e., silica-rich or silica-poor. The composition plot of silica-poor gel is near to apical (C + M + N). Therefore, it is believed that the composition return at the spots 001M and 002M is spurious, and the pit wall had silica-poor C-A-S-H gels not due to carbonation.

Impure N-A-S-H gels, called “hybrid gel” combined with some C-A-S-H components, are expressed as N-(C)-A-S-H, which occupy the intermediate region of the trend line [34]. Strictly saying, the gels in the FA/GGBS blend-based geopolymers are hybrid gels even for the C-A-S-H that should be C-(N)-A-S-H. Moreover, all the gels contained different impurities, as described in Table 3 and Table 4.

Other markers in Figure 8, representing different gels cited from Refs. [36,54,67], will be explained bellow.

##### Al_2_O_3_-(CaO + MgO)-Na_2_O Diagram

Figure 9, which was drawn by separating the Na_2_O-component from the sum of (CaO + MgO + Na_2_O) components, was used to discuss the behavior of the Na_2_O which is one of main components of the GP-liquor used. There is a clear boundary between C-A-S-H and N-A-S-H plot areas without any exception, which is the A50(C + M)50-(C + M)50N50 line in molar. This boundary agrees with the previous study [35], which was determined by the SEM-EDS technique in respect to the morphological difference between C-A-S-H and N-A-S-H gels. The scattering plots represent raw FA particles and their derivatives of N-A-S-H, as observed in Figure 8. However, Figure 9 shows that the plots for N-A-S-H gels occupy the two separate areas, which correspond to Ca(Mg)-rich N-A-S-H and Ca(Mg)-poor N-A-S-H, respectively. The composition plots of C-A-S-H distribute along (C + M)-(Ab, Ne) join, but this distribution is slightly broad, compared with Figure 8. The position of the (C + M)-(Ab, Ne) join indicates that molar ratio of Al/Na = 1 is necessary to keep charge neutrality when Al^3+^ replace Si^4+^. Some deviation from this join is due to the effect of impurities comprised in the gels or metastability of gel compositions on the way to element equilibrium.

The plots for the composition data in Refs. [36,54,67] fall into the plot area of N-A-S-H, as shown in Figure 9. The red and green crosses represent synthetic genuine N-A-S-H gels introduced in different literature [36,67]. The synthetic N-A-S-H gels, plotted as green crosses, changed to nepheline after heating at an elevated temperature [67]. These gels were plotted just on nepheline (Ne) point, as shown in Figure 9. On the contrary, in Figure 8 they were plotted only near the Ne-point, indicating that they were slightly silica-rich in composition. On the other hand, other synthetic genuine N-A-S-H gels [36], marked with red crosses, were plotted near the Ne-point, or near and parallel to the (C + M + N)-Ab join in Figure 8. In Figure 9, however, they were plotted in line on the sodium-rich side deviating from the Ab or Ne-point.

Synthetic Ca-bearing N-A-S-H [36] was plotted very near to the line of (C + M)-(Ab, Ne) in the N-A-S-H plot area, indicating Al/Na is near to 1.0 (see the large bold red cross in Figure 9). In Figure 8, this gel was plotted near the proposed border between N-A-S-H and C-A-S-H plots, and on the C-A-S-H side. Furthermore, two N-A-S-H gels formed in the sole FA-based geopolymer [54] were plotted, marked with blue diamonds, near or far from the joins, as shown in Figure 8 and Figure 9, respectively, reflecting raw FA’s characters in compositions.

Consequently, for the FA70%/GGBS30% blend-based GP, in a long term after carbonation, initial C-A-S-H and N-A-S-H gels may gradually incorporate each other in compositions, the ultimate boundary points of component equilibria on the two joins would be at the positions, EQP-1 (65, 25, 10) and EQP-2 (25, 50, 25) in two ternary diagrams, respectively.

## 4. Conclusions

In this study, we investigated the compositions and microstructures of the matrix gels of three kinds of geopolymer (GP) pastes using FA or GGBS alone or their blend as precursor, respectively, and their changes as well as new products after carbonation by the SEM/EDS and XRD techniques and the ternary diagram analysis. The following conclusions were obtained.

Regardless of carbonation, N-A-S-H gels with a flaky texture and C-A-S-H gels with a spongy texture were confirmed in sole FA, sole GGBS-based GP, respectively. The flake-like N-A-S-H gels grew on the linear alignments of FA particles with small sizes, called “beads”. However, in the FA/GGBS blend-based GP, in addition to sponge-like normal C-A-S-H gels, two types of abnormal C-A-S-H gels were found around the pop-out pits of FA particles, which were dense gels forming pit walls, and fluffy cotton-like gels covering the pit walls of FA particle residues in beads texture. The two abnormal C-A-S-H gels were judged as C-A-S-H gels rather than N-A-S-H gels.

The N-A-S-H gels in the carbonated GP using FA alone as a precursor exhibited small pores around FA particle residues. As a product of carbonation, nahcolite was identified for the FA-based GP by XRD analysis. For the GPs using GGBS alone or in a blend as a precursor, calcite is confirmed after carbonation by XRD analysis and SEM observation. After carbonation, the FA/GGBS blend-based GP had more calcite, compared to the GGBS-based GP. The carbonation resistance of C-A-S-H gel is independent of Ca content and may be influenced by its degree of compactness.

Cube-shaped calcite and small calcite grains were found in the carbonated GGBS-based GP sample. In the carbonated FA/GGBS blend-based GP, the complexes of calcite and C-A-S-H gels were generated in form of hail-like particles. The hail-like particles were found to plug the top of the pores of the spongy C-A-S-H gels.

Geopolymer gels are characterized as N-A-S-H and C-A-S-H. Combined ternary diagram analysis of SiO_2_-(CaO + MgO + Na_2_O)-Al_2_O_3_ and Al_2_O_3_-(CaO + MgO)-Na_2_O, in which oxide components of gels are determined by SEM-EDS analysis, can judge the gel type of geopolymer, as well as the products and compositional changes after carbonation. N-A-S-H and C-A-S-H gels are plotted in two separate areas divided by S75(C + M + N)25-A75(C + M + N)25 line in the S-(C + M + N)-A diagram, and A50(C + M)50-N50(C + M)50 line in the A-(C + M)-N diagram, respectively. C/N = 1.0 or (C + M)/N = 1.0 in molar can be used to distinguish between N-A-S-H and C-A-S-H gels, i.e., N-A-S-H ≤ 1.0 ≤ C-A-S-H. The distribution of plots for N-A-S-H gels is wide due to the heterogeneous compositions of raw FA particles. The C-A-S-H plots are aligned along with the apical (C + M + N)-Ab join, and the plots for the C-A-S-H gels in FA/BFS blend-based GP locate intermediate area of the join, while the plot for the complex of C-A-S-H gel and calcite is shifted toward apical (C + M + N) of the SiO_2_-(CaO + MgO + Na_2_O)-Al_2_O_3_ diagram.

## Figures and Tables

**Table 1:** Chemical compositions and physical properties of active fillers, normalized to 100% by mass.

	SiO_2_	TiO_2_	Al_2_O_3_	Fe_2_O_3_	MnO	CaO	MgO	Na_2_O	K_2_O	P_2_O_5_	SO_3_	LOI ^1^	Density (g/cm^3^)	SSA ^2^ (m^3^/kg)
FA	60.26	1.32	22.36	6.68	0.05	2.01	0.65	0.52	1.63	0.71	0.95	2.86	2.24	355
GGBS	33.54	0.55	13.99	0.33	0.20	41.72	5.32	0.24	0.24	0.04	2.47	1.40	2.89	418

[Notes] ^1^ Loss on ignition, and ^2^ Specific surface area, Blaine value.

**Table 2:** Mix proportions of GP pastes, carbonation method and specimen ID.

No.	FA: GGBS (Mass Ratio)	Non- Carbonation	Accelerated Carbonation	GP-Liquor/Filler Ratio (by Mass)
1.	100:0	N-0	C-0	0.5
2.	70:30	N-30	C-30	0.5
3.	0:100	N-100	C-100	0.5

[Notes] GP-liquor used: S/N = 1.1 in molar, density = 1.31, and concentration = 26.3% by mass.

**Table 3:** SEM-EDS analytical results of matrix gels for the non-carbonated samples, normalized to 100 in molar.

**N-A-S-H**													
Remark	Spot ^1^	SiO_2_	TiO_2_	Al_2_O_3_	Fe_2_O_3_	CaO	MgO	Na_2_O	K_2_O	P_2_O_5_	SO_3_	C/N	(C + M)/N
Beads	N-0-9	63.67	0.76	12.52	1.82	3.35	1.07	11.32	0.90	2.23	2.37	0.30	0.39
Beads	N-0-10	58.98	0.28	22.81	1.21	2.85	0.79	10.11	0.52	0.95	1.51	0.28	0.36
Beads	N-0-11	63.61	0.49	9.53	1.78	4.55	1.13	14.18	0.69	1.92	2.12	0.32	0.40
	N-0-12	58.78	0.90	24.23	1.86	3.26	1.34	5.66	0.40	1.68	1.88	0.58	0.81
	N-0-13	71.55	0.62	9.51	4.55	4.26	0.66	4.03	1.27	1.69	1.86	1.06	1.22
	N-0-14	68.91	0.85	10.00	2.46	5.07	0.90	7.12	0.85	1.53	2.32	0.71	0.84
	N-0-15	70.88	0.73	9.27	5.07	4.17	1.17	3.14	1.61	1.88	2.09	1.33	1.70
	N-0-16	69.44	0.41	8.13	1.59	3.57	1.53	10.62	0.64	1.56	2.49	0.34	0.48
	N-0-17	65.49	1.15	9.80	2.85	5.75	1.31	7.80	0.89	2.34	2.61	0.74	0.91
	N-0-18	67.41	0.52	12.78	1.79	3.77	1.41	6.88	0.72	2.06	2.65	0.55	0.75
**C-A-S-H**													
Remark	Spot ^1^	SiO_2_	TiO_2_	Al_2_O_3_	Fe_2_O_3_	CaO	MgO	Na_2_O	K_2_O	P_2_O_5_	SO_3_	C/N	(C + M)/N
Beads	N-30-1	53.31	0.59	8.39	1.09	24.31	4.95	3.16	0.31	1.35	2.55	7.69	9.26
	N-30-3	53.75	0.35	7.68	0.72	28.51	3.42	3.01	0.24	0.64	1.68	9.47	10.61
Beads	N-30-4	54.42	0.45	10.79	0.95	20.26	4.47	4.53	0.36	1.20	2.57	4.47	5.46
	N-30-5	56.80	0.57	8.04	1.02	20.72	4.00	4.22	0.45	1.10	3.07	4.91	5.86
Beads	N-30-6	53.94	0.42	10.91	0.80	19.00	4.98	5.48	0.35	1.29	2.82	3.47	4.38
Beads	N-30-7	80.64	.0.21	4.36	0.41	8.51	1.97	1.97	0.20	0.42	1.30	4.32	5.32
Beads	N-30-8	63.31	0.28	8.81	0.74	14.87	4.04	4.94	0.26	0.95	1.80	3.01	3.83
	N-30-9	56.80	0.37	8.59	0.80	19.37	4.86	4.53	0.31	0.92	3.45	4.28	5.35
Beads	N-30-10	52.96	0.36	9.43	0.88	20.56	5.40	6.28	0.22	1.39	2.52	3.27	4.13
Beads	N-30-11	55.81	0.40	10.04	0.74	18.03	5.46	5.58	0.34	1.10	2.49	3.23	4.21
	N-30-3D	54.41	0.64	4.34	1.63	27.75	5.87	4.37	0.19	0.04	0.75	6.35	7.69
	N-30-4D	57.87	0.59	5.33	1.89	23.92	4.69	4.47	0.36	0.09	0.80	5.35	6.40
Beads, Cotton	N-30-1S	59.89	0.28	5.67	3.29	20.19	6.71	3.37	0.29	0.08	0.22	5.99	7.98
Beads, Cotton	N-30-2S	59.04	0.43	5.41	2.19	21.55	4.61	5.68	0.23	0.05	0.80	3.79	4.61
Pit	N-30-5S	59.55	0.34	6.22	2.62	23.79	3.81	2.72	0.34	0.02	0.61	8.74	10.14
Pit(ring)	N-30-7S	57.22	0.41	5.36	1.45	20.95	4.57	9.16	0.22	0.04	0.62	2.29	2.79
Pit	N-30-1M	38.69	1.73	5.13	1.98	44.08	1.37	1.85	0	1.20	4.06	23.83	24.57
Pit	N-30-2M	37.45	0	4.12	2.26	46.40	0.80	1.54	0.29	2.29	4.85	30.13	30.65
Pit	N-30-3M	53.01	0.18	9.24	0.5	14.39	5.31	7.85	0.2	4.29	5.04	1.83	2.51
	N-30-4M	54.48	0.48	8.71	0.44	13.68	6.01	4.95	0.18	5.75	5.33	2.76	3.98
	N-30-5M	54.26	0.09	8.90	0.29	13.07	5.49	5.73	0.27	5.51	6.38	2.28	3.24
	N-30-6M	51.46	0.28	8.62	0.72	12.38	5.63	6.99	0.06	6.41	7.44	1.77	2.58
	N-30-7M	49.14	0.26	7.92	0.69	15.06	5.09	6.74	0.24	7.58	7.27	2.23	2.99
**C-A-S-H**													
Remark	Spot ^1^	SiO_2_	TiO_2_	Al_2_O_3_	Fe_2_O_3_	CaO	MgO	Na_2_O	K_2_O	P_2_O_5_	SO_3_	C/N	(C + M)/N
	N-100-2	37.56	0.48	7.97	0.07	26.81	15.76	8.21	0.09	1.15	1.89	3.27	5.19
	N-100-6	42.88	0.40	6.01	0.07	35.68	4.97	5.19	0.26	1.55	2.99	6.87	7.83
	N-100-7	45.17	0.12	6.87	0.08	30.24	4.86	8.85	0.04	1.43	2.35	3.42	3.97
	N-100-8	43.66	0.08	6.77	0.15	29.36	5.78	10.19	0.10	1.29	2.62	2.88	3.45
	N-100-9	42.94	0.20	6.40	0	32.43	4.53	8.95	0.17	1.46	2.93	3.62	4.13

[Notes] ^1^ First number 0 or 00 of spot codes shown in SEM-EDS images was omitted here for simplicity.

**Table 4:** SEM-EDS analytical results of matrix gels for carbonated samples, normalized to 100 in molar.

**N-A-S-H**													
Remark	Spot ^1^	SiO_2_	TiO_2_	Al_2_O_3_	Fe_2_O_3_	CaO	MgO	Na_2_O	K_2_O	P_2_O_5_	SO_3_	C/N	(C + M)/N
Beads	C-0-9	83.65	0.18	6.48	0.60	0.46	0.74	3.49	0.53	1.87	2.01	0.13	0.34
Beads	C-0-10	71.63	0.38	6.28	1.13	1.60	0.80	12.91	0.50	1.89	2.87	0.12	0.19
Beads	C-0-11	60.49	0.45	9.04	1.40	2.69	1.14	17.83	0.49	2.89	3.58	0.15	0.21
	C-0-12	66.08	0.44	8.39	2.34	3.49	0.84	10.43	0.66	2.47	4.85	0.33	0.42
	C-0-13	67.73	0.79	9.80	2.93	5.92	1.23	6.18	0.91	1.29	3.23	0.96	1.16
	C-0-14	65.27	0.42	7.97	1.61	4.74	1.33	12.61	0.56	2.14	3.35	0.38	0.48
	C-0-15	67.85	1.31	6.78	4.42	8.28	0.50	4.30	1.26	1.78	3.51	1.93	2.04
**C-A-S-H**													
Remark	Spot ^1^	SiO_2_	TiO_2_	Al_2_O_3_	Fe_2_O_3_	CaO	MgO	Na_2_O	K_2_O	P_2_O_5_	SO_3_	C/N	(C + M)/N
	C-30-6	52.77	0.49	10.22	0.82	23.26	4.38	5.90	0.46	0.13	1.56	3.94	4.68
	C-30-7	53.76	0.65	8.98	1.17	18.15	4.76	11.07	0.44	0.02	1.00	1.64	2.07
Hail ^2^, CR ^3^	C-30-1F	29.76	0.34	5.02	0.53	48.42	3.95	7.08	0.21	2.02	2.68	6.84	7.40
Hail, CR	C-30-2F	42.29	0.44	7.02	0.8	34.60	3.68	4.72	0.31	2.75	3.39	7.33	8.11
Hail, CR	C-30-3F	42.39	0.58	7.64	0.46	32.27	3.65	6.18	0.42	3.05	3.36	5.22	5.81
Hail, CR	C-30-4F	46.94	0.53	7.00	0.88	31.89	3.85	4.24	0.42	1.74	2.51	7.52	8.43
	C-30-5F	52.84	0.53	7.41	1.00	25.29	3.88	3.86	0.39	1.67	3.12	6.55	7.56
Hail	C-30-1J	51.81	0.28	7.50	0.77	21.85	4.80	7.62	0.34	2.00	3.02	2.87	3.50
Hail	C-30-2J	52.94	0.52	8.59	0.64	21.32	5.50	5.68	0.40	1.45	2.96	3.75	4.72
Hail, CR	C-30-3J	47.99	0.59	7.68	0.86	28.37	4.52	4.39	0.34	2.07	3.19	6.46	7.49
**C-A-S-H**													
Remark	Spot ^1^	SiO_2_	TiO_2_	Al_2_O_3_	Fe_2_O_3_	CaO	MgO	Na_2_O	K_2_O	P_2_O_5_	SO_3_	C/N	(C + M)/N
	C-100-6	45.61	0.51	7.23	0	29.86	7.21	6.13	0.16	1.21	2.08	4.87	6.05
	C-100-7	47.10	0.22	7.08	0	31.18	5.26	5.68	0.22	1.26	2.01	5.49	6.42
	C-100-8	43.61	0.36	6.88	0.04	30.87	7.94	7.02	0.04	1.16	2.09	4.40	5.53
	C-100-9	44.68	0.22	6.67	0.04	31.16	5.50	8.09	0.32	1.08	2.23	3.85	4.53

[Notes] ^1^ First number 0 or 00 of spot codes shown in SEM-EDS images was omitted here for simplicity, ^2^ Hail-like particle, and CR ^3^: spot with composition return due to carbonation, referring to the description in Section 3.2.3.

## Data Availability

Data are contained within the article.
